# Colorectal cancer surgery in selected nonagenarians is relatively safe and it is associated with a good long-term survival: an observational study

**DOI:** 10.1186/s12957-020-01895-8

**Published:** 2020-06-03

**Authors:** Cristina Roque-Castellano, Roberto Fariña-Castro, Eva María Nogués-Ramia, Manuel Artiles-Armas, Joaquín Marchena-Gómez

**Affiliations:** 1 Department of General Surgery, Hospital Universitario de Gran Canaria Dr. Negrín, Universidad de Las Palmas de Gran Canaria, Las Palmas de Gran Canaria, Spain; 2 Department of Anesthesiology, Hospital Universitario de Gran Canaria Dr. Negrín, Universidad de Las Palmas de Gran Canaria, Las Palmas de Gran Canaria, Spain; 3 Department of General and Digestive Surgery, Hospital Universitario de Gran Canaria Dr. Negrín, Universidad de Las Palmas de Gran Canaria, Las Palmas de Gran Canaria, Spain

**Keywords:** Colorectal cancer, Nonagenarians, Comprehensive Complication Index, Mortality, Long-term survival

## Abstract

**Background:**

Advanced age is a risk factor for colorectal cancer, and very elderly patients often need to be surgically treated. This study aimed to analyze the outcomes of a cohort of nonagenarian patients operated on for colorectal cancer.

**Methods:**

Observational study conducted on a cohort of 40 nonagenarian patients, who were treated surgically for colorectal cancer between 2000 and 2018 in our institution. Clinical data, ASA score, Charlson Comorbidity Index, Surgical Mortality Probability Model, tumor characteristics, and nature and technical features of the surgical procedure, were recorded. The Comprehensive Complication Index (CCI) and survival time after the procedure were recorded as outcome variables. Univariate and multivariate analyses were performed in order to define risk factors for postoperative complications and long-term survival.

**Results:**

Out of the 40 patients, 13 (32.5%) were men, 27 (67.5%) women, and mean age 91.6 years (SD ± 1.5). In 24 patients (60%), surgery was elective, and in 16 patients (40%), surgery was emergent. Curative surgery with intestinal resection was performed in 34 patients (85%). In 22 patients (55%), intestinal continuity was restored by performing an anastomosis. The median CCI was 22.6 (IRQ 0.0–42.6). Operative mortality was 10% (4 patients). Cumulative survival at 1, 3, and 5 years was 70%, 47%, and 29%, respectively. In multivariate analysis, only the need for transfusion remained as an independent prognostic factor for complications (*p* = 0.021) and TNM tumor stage as a significant predictor of survival (HR 3.0, CI95% 1.3–7.2).

**Conclusions:**

Colorectal cancer surgery is relatively safe in selected nonagenarian patients and may achieve long-term survival.

## Introduction

People worldwide are living longer. Today, most people can expect to live into their sixties and beyond and life expectancy is gradually increasing. There is more than a 50% probability that by 2030, the national female life expectancy will break the 90-year barrier, a level that was deemed unattainable by some at the turn of the twenty-first century [1].

Age is a major risk factor for colorectal cancer (CRC), being the third most frequent in the adult population [2]. The incidence of CRC increases with age, with a median age of diagnosis at about 70 years [3]. Specifically, CRC accounts for more than one fifth of the new cases of cancer in people aged 90 years or older. In fact, in women aged 90 or older, the most common sites of cancers are colorectal, breast, and lymphoma/leukemia. In nonagenarian men, CRC is the most frequently observed after prostate cancer [4]. Cancer survival is largely determined by the receipt of a potentially curative treatment, which in the case of CRC is mainly surgery. In these patients, treatment options will depend essentially on the stage of the disease and the health status of patients, which is usually determined by their age, comorbidity, and frailty [5].

It is necessary to highlight that the exclusion of older patients from randomized clinical trials has resulted in a lack of evidence-based guidelines [3]. In the past, nonagenarians with CRC were treated less aggressively because of their age and comorbidity. There was a certain reluctance to submit these patients to a surgical procedure, and some people even considered that no treatment was the best treatment option in this population [6]. Nevertheless, improvements in perioperative management and the development of laparoscopic colorectal surgery have provided great postoperative benefits in the elderly, especially in those older and debilitated. Actually, many of these studies that have shown favorable results regarding morbimortality have reported mainly octogenarian patients [7], being few the series focused on nonagenarian patients.

Only five series have been reported in which nonagenarians with CRC have been exclusively included and most of them with a small number of patients [8–12]. Some other studies have differentiated the characteristics of nonagenarians but in the context of large series that mainly include octogenarians [2, 13, 14].

The aim of this study was to analyze postoperative and long-term outcomes of a cohort of nonagenarians who underwent colorectal cancer surgery in our center, as well as to analyze the factors related to postoperative complications measured by the Comprehensive Complication Index (CCI) and long-term survival.

## Methodology

### Study design and participants

An observational and longitudinal study was conducted on a cohort of 40 nonagenarian patients who were consecutively treated surgically for CRC between 2000 and 2018 in our institution. The setting was a tertiary care hospital with a catchment population of approximately 400,000 inhabitants. Exclusion criteria included the non-operated nonagenarian patients and those whose clinical records or follow-up was incomplete or not available. The number and characteristics of the non-operated patients were not collected. The data were gathered from the hospital computerized diagnostic coding database and the review of all identified medical records.

### Method

A complete clinical history and a preoperative anesthetic assessment were performed on patients who underwent elective surgery. The diagnosis of CRC was carried out by colonoscopy and biopsy. An extension study utilizing thoracoabdominal computed tomography was also performed. The day before surgery, anterograde colon mechanical preparation was implemented as well as preoperative antibiotic prophylaxis 30–60 min before the surgical intervention. Obviously, the patients who required emergency surgery did not meet the requirements mentioned above as colonoscopy, biopsy, and preoperative preparation, except for the antibiotic prophylaxis which was administered in all cases.

The following data were recorded:

### Clinical data

The clinical data recorded were age, sex, the American Society of Anesthesiology Physical Status Score (ASA-PS) [15] categorized as ASA-PS I-II vs ASA-PS III-IV, patient comorbidity measured by the Charlson Comorbidity Index [16] and Surgical Mortality Probability Model (S-MPM) [17].

The Charlson Comorbidity Index was calculated preoperatively in each patient using an electronic application [18]. This score includes 19 medical conditions assigned values of 1, 2, 3, or 6, with totals ranging from 0 to 37 points. In general, the absence of comorbidity is considered 0–1 point, low comorbidity 2 points, and high comorbidity ≥ 3 points. In this study, the Charlson Index was not adjusted for age, or for the prevalence of AIDS, as proposed Zavascki and Fusch [19], as there were no cases of this in the study population.

The Surgical Mortality Probability Model (S-MPM) [17] is a simple risk index for all-cause 30-day mortality for noncardiac surgery. The 9-point S-MPM was derived empirically and includes three risk factors: ASA physical status, emergency status, and surgery risk class. Patients with ASA physical statuses I, II, III, IV, or V were assigned either 0, 2, 4, 5, or 6 points, respectively; intermediate or high-risk procedures were assigned 1 or 2 points, respectively; and emergency procedures were assigned 1. The S-MPM score was categorized as class I (0–4 points), class II (5–6 points), and class III (7–9 points).

### Surgical variables

Tumor location (right side vs left side), surgical site where the surgery was performed (colon or rectum), nature of the procedure (elective vs emergency surgery), intent of the surgical procedure (curative vs palliative surgery), performance of anastomosis, and the need for perioperative blood transfusion were the surgical variables. Emergency surgery was defined as surgery within 24 h of admission. Curative surgery was defined as macroscopically complete resection without invasion of the surgical margins at histological examination. Palliative surgery was defined as a surgical procedure designed to alleviate cancer-related symptoms and to prevent the appearance of complications. Based on the need for at least one red blood cell unit transfusion during or immediately before or after the procedure, the patients were classified as transfused or not transfused. The decision to transfuse was based on a liberal transfusion strategy, generally in patients with a hemoglobin concentration ≤ 9 g/dL.

### Postoperative complications

Postoperative complications were recorded and graded according to the Comprehensive Complication Index (CCI) [20]. This index is a novel metric of postoperative morbidity, integrating with a single formula, all complications by severity, ranging from 0 (uneventful course) to 100 (death) [21]. The scale represents an improvement on the Clavien-Dindo classification in terms of its association with clinical results. For analysis purposes, the variable was evaluated in two different situations: (a) no complications (CCI = 0) vs. complications (CCI = 1) and (b) as a continuous variable (CCI from 0 to 100).

### Operative mortality

It was defined either as any death occurring within 30 days of surgery or any later death that was considered to be a direct consequence of a postoperative complication.

### Cancer stage (TNM)

Staging according to the 8th edition of the American Joint Committee on Cancer Staging was collected and categorized from stage I to stage IV.

### Long-term survival

It was considered as the period between the performance of the surgical procedure and death or the date of the last follow-up observation before the analysis if the subject was still alive. The mean follow-up of the cohort was 35 months, and the median follow-up was 21 months.

## Statistical analysis

Data were analyzed using the statistical package SPSS 17.0 for Windows (SPSS, Chicago, IL, USA). First, a descriptive study of the sample was carried out. Categorical variables were expressed as frequencies and percentages, and the numerical variables by the mean and standard deviation or the median and interquartile range. The survival curves were constructed using the Kaplan-Meier method.

Next, a univariate analysis of both postoperative complications and long-term survival was performed. The sample was also separated into two groups: patients undergoing elective and curative surgery versus patients undergoing urgent and palliative surgery. Both groups were compared. The chi-square test or Fisher’s test was used to compare categorical data. For continuous variables, the Mann-Whitney *U* test, or the Kruskal-Wallis test for nonparametric distributions, was used as appropriate. Linear regression was also used for comparing two continuous variables. Multivariate linear regression was performed on those variables associated with CCI in the univariate analysis to determine their prognostic significance.

In survival analysis, the differences between the survival curves were tested by the log-rank test or by the Tarone-Ware test as appropriate. The relative prognostic significance of the variables in predicting overall survival was assessed using multivariate Cox proportional hazards regression analysis. Hazard ratios were also calculated as association measurements using a Cox regression model. Statistical significance was defined as *p* < 0.05.

## Results

### Descriptive analysis

Out of the 40 patients in the cohort, 13 (32.5%) were men and 27 (67.5%) were women (*p* < 0.001), with a mean age of 91.6 years (SD ± 1.5 years). Most of the patients lived at home with at least a relative and/or caregiver. Only 3 patients (7.5%) were institutionalized. In 24 patients (60%), surgery was elective, and in 16 patients (40%), surgery was emergent.

Regarding risk scales, only 8 patients (20%) were classified as ASA I-II, and 32 patients (80%) were classified as ASA III-IV. Five patients (13%) were S-MPM grade I, 27 (67%) grade II, and 8 (20%) grade III. According to the Charlson Comorbidity Index, 8 patients (20%) had no comorbidity, 11 (28%) had low comorbidity, and 21 (52%) had high morbidity.

The neoplasm was located in the right colon in 19 cases (47.5%), in the transverse colon in 3 cases (7.5%), in the left colon in 14 cases (35%), and in the rectum in 4 cases (10%). Curative surgery was performed in 34 patients (85%), and palliative surgery was performed in 6 patients (15%), including 4 cases of rectal cancer in whom neoadjuvant chemoradiation therapy was not applied, and 2 patients with liver metastases. In all patients undergoing curative surgery, intestinal resection was performed. In 22 patients (55%), intestinal continuity was restored by performing an anastomosis, while in 18 cases (45%) no anastomosis was carried out. The anastomosis was performed in all patients in whom a right colectomy was performed except in one case, and no anastomosis was performed in all patients operated on for neoplasms in the left side of the colon except in 3 cases. Details of the surgical procedures are shown in Table 1.

**Table 1:** Operation types

Operation	*N* (%)
Right colectomy	18 (45%)
Extended right colectomy	1 (2.5%)
Subtotal colectomy	1 (2.5%)
Transversectomy	2 (5.0%)
Sigmoidectomy	3 (7.5%)
Hartmann’s procedure	9 (22.5%)
Trans-anal excision	2 (5.0%)
Defunctioning stoma	4 (10.0%)

In seven cases (17.5%), a laparoscopic approach was carried out to perform the surgical procedure.

Perioperatively, 19 patients (25.2 %) received at least one red blood transfusion. The need for transfusion was not significantly associated with comorbidity measured by the Charlson Index (*p* = 0.405), but it was associated with the location of the neoplasm (*p* = 0.001). Patients with malignancies of the right colon required significantly more transfusions than in patients with malignancies of the left colon (69.6% vs 17.6%).

With regard to cancer staging, 28 patients (70%) had non-disseminated disease (stages I–II), 8 patients (20%) were stage III, and 4 patients (10%) were stage IV. All stage IV patients had rectal cancer and underwent palliative surgery.

Only 12 patients (30%) were free of postoperative complications. The remaining 70% had some type of complication, although many of them were minor complications. The mean CCI was 28.7 (± 30.0), and the median CCI was 22.6 (IQR 0.0–42.6). Most patients (57.5%) had a CCI score ≤ 30 (either no complications or minor complications), while 42.5% had serious complications including death. It was remarkable that all patients who reached a CCI score ≥ 60, finally died.

In 20 patients (50%), *surgical* postoperative complications were registered, with postoperative ileus (19 cases) and wound infection (5 cases) being the most frequently observed. In the 22 patients in whom a primary anastomosis was performed, no suture dehiscence was recorded. Regarding *non-surgical* complications, which occurred in 18 patients (45%), renal failure (16 cases), confusional syndrome (11 cases), and non-fatal infections of different origins (8 cases), were the most frequently observed.

Operative mortality was 10% (4 patients), with three of them being operated on in an emergency setting. In the group of elective surgeries, only 1 patient (4.2%) died. Causes of death were multiorgan failure (2 cases), cardiogenic shock (1 case), and respiratory failure (1 case). Mean stay was 12.5 days (± 6.5 days), and there was no need for reoperation in any patient.

**Fig. 1:**
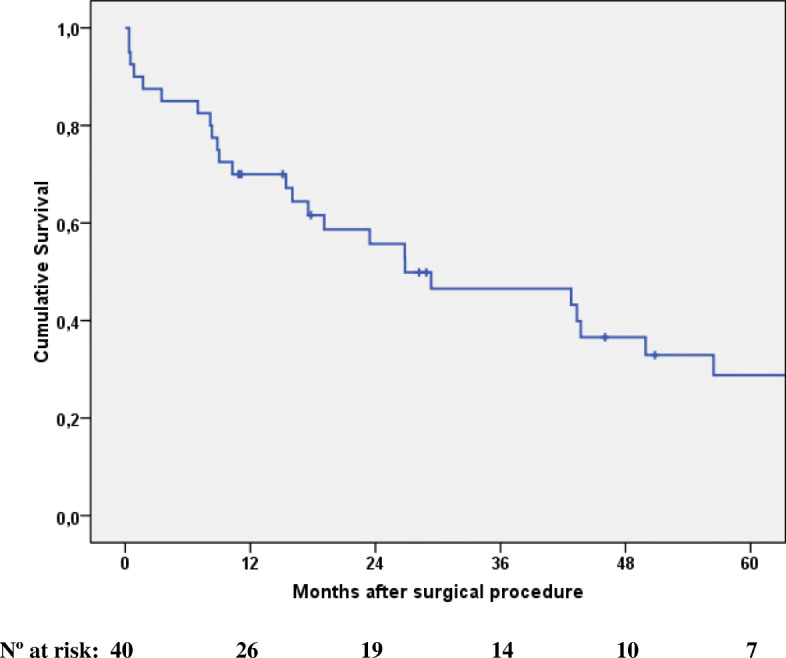
Overall survival function of the nonagenarian patients operated on by colorectal cancer (Kaplan-Meier)

At the end of the follow-up, 11 patients (27.5%) remained alive. Median survival was 26.9 months (IQR 84.4–8.8). Cumulative survival at 1, 3, and 5 years was 70%, 47%, and 29%, respectively (Fig. 1). The survival of the four patients with rectal cancer ranged from 8 months to 2.4 years, and only one of them remained alive at the end of follow-up.

All patients that were discharged returned to the same level of care before the admission at the hospital, but we could not gather information on either their postoperative functional status or their quality of life after the surgical procedure.

### Comparative analysis of the two groups

In 21 patients (52.5%), elective and curative surgeries were performed, while in 19 patients (47.5%) emergency and/or palliative surgery were carried out. The differences between both groups are shown in Table 2. Significantly fewer anastomoses were performed in patients in whom palliative or emergency surgery was performed (*p* < 0.001). The median long-term survival was also significantly lower (*p* = 0.034) in these patients.

**Table 2:** Comparative analysis between the group of patients in whom elective and curative surgery was performed, and the group of patients in whom palliative and/or emergency surgery were carried out

	Total	Elective and curative surgery 21 (52.5%)	Palliative and/or emergency surgery 19 (47.5%)	*p*
Age (mean ± SD)	91.6 (± 1.5)	91.5 (± 1.6)	91.7 (±1.4)	0.737
Gender				0.427
Man	13 (32%)	8 (38.1%)	5 (26.3%)	
Woman	27 (68%)	13 (61.9%)	14 (73.7%)	
Institutionalized	3 (7.5%)	0 (0.0%)	3 (15.8%)	0.098
ASA				0.874
I–II	8 (20%)	4 (19.0%)	4 (21.1%)	
III–IV	32 (80%)	17 (81.0%)	15 (78.9%)	
S-MPM				0.031
I	5 (13%)	3 (14.3%)	2 (10.5%)	
II	27 (67%)	18 (85.7%)	9 (47.4%)	
III	8 (20%)	0 (0.0%)	8 (42.1%)	
Charlson Comorbidity Index				0.065
No	8 (20%)	2 (9.5%)	6 (31.6%)	
Low	11 (28%)	5 (23.8%)	6 (31.6%)	
High	21 (52%)	14 (66.7%)	7 (36.8%)	
Neoplasm location				0.061
Right side^a^	23 (58%)	15 (71.4%)	8 (42.1%)	
Left side	17 (42%)	6 (28.6%)	11 (57.9%)	
Anastomosis				< 0.001
No	18 (45%)	3 (14.3%)	15 /78.9%)	
Yes	22 (55%)	18 (85.7%)	4 (21.1%)	
Transfusion				0.055
No	21 (53%)	8 (38.1%)	13 (68.4%)	
Yes	19 (47%)	13 (61.9%)	6 (31.6%)	
TNM stage				0.112
I–II	28 (70%)	17 (81.0%)	11 (57.9%)	
III–IV	12 (30%)	4 (19.0%)	8 (42.1%)	
CCI (median–IQR)	22.6 (0.0–42.6)	22.6 (10.5–34.1)	12.2 (0.0–54.9)	0.748
Operative mortality	4 (10.0%)	1 (4.8%)	3 (15.8%)	0.246
Median survival in months (IQR)	26.8 (84.4–26.5)	49.9 (99.8–19.1)	17.5 (29.3–8.4)	0.034

^a^Including transverse colon location

### Uni- and multivariate analysis

Univariate analysis of postoperative complications measured by the CCI is shown in Table 3. Age (*p* = 0.014), palliative surgery (*p* = 0.002), and need for transfusion (*p* < 0.001) were associated with CCI—palliative surgery, paradoxically, in a protective sense. By adjusting the three variables in a linear regression model, only transfusion remained as an independent prognostic factor for complications (*p* = 0.021) (Table 4).

**Table 3:** Univariate analysis of risk factors for CCI (Comprehensive Complications Index) 0/1 (no complications vs. complications), and CCI 0–100 score

Risk factors	Total *n* = 40	Complications		CCI 0/1 (*p* value)	Median CCI (IQR) 22.6 (0.0–42.6)	CCI 0–100 (*p* value)
		No (*n* = 12) (30%)	Yes (*n* = 28) (70%)			
Age (mean ± SD)	91.6 (± 1.5)	91.1 (± 1.2)	91.8 (± 1.6)	0.110	–	0.014^b^
Gender						
Man	13 (32%)	3 (25.0%)	10 (35.7%)	0.507	22.6 (4.4–77.5)	0.391
Woman	27 (68%)	9 (75.0%)	18 (64.7%)		22.6 (0.0–36.2)	
ASA						
I–II III–IV	8 (20%) 32 (80%)	3 (25.0%) 9 (75.0%)	5 (17.9%) 23 (82.1%)	0.605	15.7 (0.0–39.2) 23.4 (0.0–43.7)	0.475
S-MPM						
I	5 (13%)	2 (16.7%)	3 (10.7%)	0.673	22.6 (0.0–61.3)	0.390
II	27 (67%)	8 (66.7%)	19 (67.9%)		22.6 (0.0–32.0)	
III	8 (20%)	2 (16.7%)	6 (21.4%)		45.6 (2.2–89.7)	
Charlson Comorbidity Index						
No	8 (20%)	2 (5.0%)	6 (21.4%)	0.631	33.7 (2.2–56.7)	0.698
Low	11 (28%)	5 (41.7%)	6 (21.4%)		8.7 (0.0–100)	
HIgh	21 (52%)	5 (41.7%)	16 (57.1%)		22.6 (6.1–34.1)	
Neoplasm location						
Right side^a^	23 (58%)	3 (25.0%)	20 (71.4%)	0.006	30.0 (20.9–50.7)	0.015
Left side	17 (42%)	9 (75.0%)	8 (28.6%)		0.0 (0.0–26.7)	
Type of surgery						
elective	24 (60%)	7 (58.3%)	17 (60.7%)	0.919	22.6 (0.0–31.7)	0.576
emergency	16 (40%)	5 (41.7%)	11 (39.3%)		23.4 (0.0–57.8)	
Intent of surgery						0.002
Curative	34 (85%)	7 (58.3%)	27 (96.4%)	0.006	27.5 (8.7–47.3)	
Palliative	6 (15%)	5 (41.7%)	1 (3.6%)		0.0 (0.0–3.1)	
Anastomosis						
No	18 (45%)	8 (66.7%)	10 (35.7%)	0.070	10.5 (0.0–40.9)	0.325
Yes	22 (55%)	4 (33.3%)	18 (64.3%)		23.4 (17.9–45.1)	
Transfusion						
No	21 (53%)	12 (100.0%)	9 (32.1%)	< 0.001	0.0 (0.0–21.8)	< 0.001
Yes	19 (47%)	0 (0.0%)	19 (67.9%)		32.0 (24.2–54.9)	
TNM stage						
I–II	28 (70%)	8 (66.7%)	20 (71.4%)	0.763	22.6 (0.0–36.2)	0.493
III–IV	12 (30%)	4 (33.3%)	8 (28.6%)		26.7 (0.0–88.7)	

^a^ Including transverse colon location

^b^Linear regression

**Table 4:** Multivariate analysis (linear regression) of risk factors associated with Comprehensive Complication Index

	Unstandardized Coefficients		Standardized Coefficients	*t*	*p*	95%CI for *B*
	*B*	Std. error	Beta			
Constant	− 504.97	253.44	–	− 1.99	0,054	− 1018.9–9.02
Age	5.55	2.77	0.274	2.00	0.053	− 0.07–11.17
Palliative surgery	17.68	11.97	0.213	1.48	0.148	− 6.59–41.95
Transfusion	21.29	8.83	0.359	2.41	0.021	3.38–39.21

*B* regression coefficient, *t* test statistic, *CI* confidence interval

Univariate analysis of long-term survival is shown in Table 5. S-MPM score (*p* = 0.022, HR 2.4; CI95% 1.1–5.2), emergency surgery (*p* = 0.022, HR 2.4; CI95% 1.1–5.1), and TNM stage (*p* = 0.003, HR 3.2; CI95% 1.4–7.2) were associated with a long-term survival. The TNM tumor stage was the only variable that remained as a significant predictor of survival in multivariate analysis (HR 3.0, CI95% 1.3–7.2) (Table 6).

**Table 5:** Univariate analysis of long-term survival

	Total (*n* = 40)	Alives (*n* = 11) (28%)	Deaths (*n* = 29) (72%)	*p*	HR (95%CI)
Age (mean ± SD)	91.6 (± 1.5)	91.5 (± 1.1)	91.7 (± 1.6)	0.310	1.1 (0.9–1.4)
Gender					
Man	13 (32%)	3 (23%)	10 (77%)	0.134	0.6 (0.2–1.2)
Woman	27 (68%)	8 (30%)	19 (70%)		
ASA					
I–II	8 (20%)	4 (50%)	4 (50%)	0.261	1.8 (0.6–5.3)
III–IV	32 (80%)	7 (22%)	25 (78%)		
S-MPM					
I	5 (13%)	2 (40%)	3 (60%)	0.022	2.4 (1.1–5.2)
II	27 (67%)	8 (30%)	19 (70%)		
III	8 (20%)	1 (13%)	7 (87%)		
Charlson Comorbidity Index					
No	8 (20%)	3 (38%)	5 (62%)	0.787	0.9 (0.5–1.4)
Low	11 (28%)	2 (18%)	9 (82%)		
High	21 (52%)	6 (29%)	15 (71%)		
Neoplasm location					
Right side*	23 (58%)	7 (30%)	16 (70%)	0.536	1.3 (0.6–2.7)
Left side	17 (42%)	4 (24%)	13 (76%)		
Type of surgery					
Elective	24 (60%)	8 (33%)	16 (67%)	0.022	2.4 (1.1–5.1)
Emergency	16 (40%)	3 (19%)	13 (81%)		
Intent of surgery					
Curative	34 (85%)	9 (27%)	25 (73%)	0.800	0.9 (0.3–2.6)
Palliative	6 (15%)	2 (33%)	4 (67%)		
Anastomosis					
No	18 (45%)	5 (28%)	13 (72%)	0.099	0.5 (0.2–1.1)
Yes	22 (55%)	6 (27%)	16 (73%)		
Transfussion					
No	21 (53%)	7 (33%)	14 (67%)	0.556	1.3 (0.6–2.6)
Yes	19 (47%)	4 (21%)	15 (79%)		
TNM stage					
I–II	28 (70%)	11 (39%)	17 (61%)	0.003	3.2 (1.4–7.2)
III–IV	12 (30%)	0 (0%)	12 (100%)		

*HR* hazard ratio

*Including transverse colon location

**Table 6:** Multivariate analysis (Cox regression) of risk factors associated with a long-term survival

	*B*	SE	Wald	*p*	HR	95%CI for HR
S-MPM	0.381	0.401	0.906	0.341	1.46	0.67–3.21
Emergency surgery	0.454	0.468	0.939	0.333	1.57	0.63–3.94
TNM stage	0.963	0.436	4.886	0.027	2.62	1.12–6.15

*B* regression coefficient, *SE* standard error, *Wald* test statistic, *HR* hazard ratio, *CI* confident interval

## Discussion

The elderly are already the most important group in medical oncology practice. The predictions of aging about this population allow us to foresee that in this age sector, cancer and its treatment will be a first-rate health problem in a few years [1]. The choice of treatment should be based on clinical status, tumor location, comorbidity, and frailty of the patients. In this age sector, this process must be highly individualized since chronological age is not always reflected in biological age.

This study demonstrates that, as is the case with younger patients, a certain number of selected nonagenarian patients with CRC should be considered for curative treatment. Although the postoperative complication rate was high (70%), the median CCI (22.6 points) was not so elevated, and the outcomes obtained in terms of operative mortality and long-term survival were acceptable, especially with elective surgery. Global operative mortality was 10% (4 patients), with the three of them being operated on in an emergency setting. Elective surgery mortality was 4.2%. These results are in accordance with other authors [8, 10, 12, 22], who have reported mortality rates oscillating between 2.1% [8] and 23% [9].

Concerning long-term outcomes, the median overall survival time after colorectal surgery was 26.9 months, which was quite higher than other studies [11] and similar to the 23.92 months reported by Chen et al. [12]. However, the 1-year overall survival rate of 70% reported here is slightly inferior to the 82.6% rate published by Schlichtemeier et al. [10], the series with the best long-term results. In the present study, in line with those published by other authors [8], one third of nonagenarian patients achieved a cumulative survival of 5 years after surgery.

Based on these results, we can support that surgical management of CRC in nonagenarians is associated with acceptable rates of morbidity, mortality, and long-term survival. Therefore, this population should not be denied definitive surgical intervention in both the elective and emergent setting [11], even though emergency surgery is subject to high mortality.

Age has long been considered one of the most important risk factors for postoperative adverse events [3, 8]. The limits of the functional reserve of the organs and tissues in the elderly patient are very narrow and are often exceeded clearly during the perioperative period. In fact, in our series, age was statistically associated with postoperative complications measured by CCI. However, age lost its significance in multivariate analysis and it was not also related to long-term survival. This suggests that other conditions must also be taken into account in predicting worse outcomes in these patients.

Frailty has been proposed as a good predictor of postoperative complications in the elderly patient undergoing a major gastrointestinal procedure [23], but this variable could not be collected due to the retrospective nature of our study. Comorbidity measured by the Charlson Comorbidity Index in the general population has been related to anastomotic leak, postoperative complications and death in Chinese patients [24], but there are not published data focused on this topic specifically in nonagenarians. In a previously reported study [25], we found that ASA score and emergency surgery were the most significant factors for operative mortality in a general nonagenarian population, but these variables lost their predictive value for postoperative complications in this subgroup of nonagenarian patients operated on for CRC. Other authors [13] have also reported than older age, higher ASA score, anemia, and lower serum albumin increased postoperative complications. Probably because of the small sample size, these variables and other contributing factors, such as surgical risk according to S-MPM score, and even emergent surgery, were not related to CCI in the present series.

The stage of the disease also did not contribute to raising the morbidity rate, contrary to what was published by other authors [8]. In our series, in those patients who underwent surgery with advanced stages, palliative procedures were only performed. These palliative procedures were accompanied by a few postoperative complications in this very elderly population. However, tumor extension was strongly related to long-term survival.

In this specific group, only the need for blood transfusion was an independent prognostic factor for postoperative complications. This finding could be considered predictable, since the need for blood transfusion would reflect per se the existence of intraoperative complications in many cases. However, in most of our patients, the indication for transfusion was not operative blood loss. Anemia is quite frequently diagnosed in older individuals and constitutes a complex problem. Nutritional deficiency anemias, bleeding anemias, secondary anemias to chronic inflammation status or chronic kidney disease, clonal anemias, and unexplained anemias, are common in the elderly [26]. Several studies have linked transfusions with many negative outcomes including death in older patients [27, 28]. Ferraris et al. [29] analyzed 8728 non-vascular thoracic operations in patients from 173 hospitals. They found that transfusion of 1 or 2 units of red blood cells increased the risk of composite morbidity, pulmonary complications, systemic sepsis, wound complications, and postoperative length of stay as compared with those who did not receive transfusions. It is not known what is really dangerous, the anemia or the transfusion itself [30], but what is remarkable is that in nonagenarians, these undesirable effects may become even more evident.

There are no previous reports of postoperative morbidity after colorectal surgery using the CCI in these elderly patients. Most of the reported postoperative complications are based on the Clavien-Dindo classification, which considers only the most serious event and underestimates postoperative morbidity as a whole [31]. Previous studies have described relatively high rates of morbidity in the elderly, except Yag et al. [8] which reported 29.2%, which was quite lower than our 70%. This may be related to the proportion of elective patients included in this last series, which was much higher than other studies (79.2%) and may reflect the low perioperative mortality and morbidity rates. Our cohort had more emergent surgeries, and it is remarkable that all kinds of complications, even minor adverse events, were collected. It is well known that the postoperative complication rate is higher in elderly patients who underwent emergent open surgeries, ranging from 27.6 to 81% [12]. Therefore, it would be advisable to avoid emergency surgery as much as possible and to offer therapeutic alternatives, such as the placement of colonic stents. This would improve the clinical and nutritional status of patients prior to elective surgery, which should benefit these patients [2].

The most frequent complication in the present study was postoperative ileus, which is quite frequent in this age group. In other series, postoperative confusion prevails [22], which is often difficult to assess and whose actual frequency of presentation can be masked in the surgical setting. Respiratory complications, renal failure, and surgical site infection have also been reported as frequent complications in nonagenarians [11].

In the present study, the procedure most frequently performed was right colectomy followed by the Hartmann procedure, which was in line with other series [8, 11, 22]. Available studies suggest that tumors are more likely to be right-sided in nonagenarian patients [2, 10]. Right colectomy seems to be less aggressive and better tolerated by very elderly people. However, we had more complications in patients operated on for right-sided lesions than in patients with left-sided lesions. Despite the fact that no anastomotic leak was observed, it should be noted that many of these patients with right side lesions were preoperatively anemic and hypoproteinemic. They required significantly more transfusions, which probably made them more susceptible to all kinds of postoperative complications.

There was also better survival in patients with right-sided tumors, but it did not reach statistical significance. Regarding the lesions in a low rectal location, no patient underwent radical surgery in our series. Also, they were not given neoadjuvant or adjuvant chemoradiation. Only local conservative treatment was applied, obtaining acceptable results in terms of long-term survival for the age.

In a large cohort of older patients operated on in California and reported by Kunitake et al. [2], an abdominoperineal resection was performed only in 16.4% of nonagenarians. In another series with less patients [8], 2 of 48 nonagenarians were submitted to this procedure. It is worth noting that Kunitake et al. [2] reported a 0.7% rate of sigmoidectomies vs. a 45.5% rate of rectal anterior resection procedures in the subgroup of nonagenarians. This circumstance suggests that the term “anterior resection of the rectum” could be preferably used in nonagenarian patients with cancer of the rectosigmoid junction or even sigmoid colon.

Anastomosis should be considered a safe surgical procedure in nonagenarian patients, at least in right colectomies. Despite having performed 18 anastomoses on the right colon and 3 on the left colon, no leaks were found. These results are consistent with those published by other authors in the few series reported that address detailed information on colorectal surgery in nonagenarians [11, 22]. Only one anastomosis leak after a right colectomy was recorded by Yap et al. [8], without mortality. Thus, surprisingly, the rate of anastomosis leakage is very low or nonexistent. These results are indicating that we should not contraindicate an anastomosis only considering age as a possible factor related to anastomotic leakage.

Laparoscopic surgery is considered an extremely useful treatment for very old patients because it has a low risk of postoperative complications, even in the presence of pre-existing diseases [14].

Yap et al. [8] reported a laparoscopic-assisted operation in 41.7% of their patients with good outcomes. They found that patients undergoing open surgery were more likely to have perioperative complications than patients undergoing minimally invasive surgery, and the mortality rate was 0%. In the present study, only 17% of patients were operated on using a laparoscopic approach, with no deaths, although this percentage has been increasing in recent years. This confirms that, even in patients > 90 years old, laparoscopic surgery for colorectal cancer is safe. Moreover, laparoscopy seems to improve postoperative outcomes in the elderly as compared with younger patients [32].

On the other hand, there are few studies that evaluate the outcomes in terms of toxicity and survival among nonagenarian patients with CRC treated with chemotherapy or concurrent chemoradiation therapy. Reddy et al. [33] reported that despite the high rate of treatment toxicity, selected octogenarian and nonagenarian patients could benefit from chemotherapy. However, in this study, which does not distinguish between octogenarians and nonagenarians, the exact role that chemotherapy could play in nonagenarian patients is not clear. In other studies [22], no patients in this age group received adjuvant therapy.

Compared with other age groups, it is evident that most surgeons are considerably less aggressive with these older patients in the presence of metastatic disease [10]. Nevertheless, it has been an important advance to not systematically deny colorectal cancer surgery in nonagenarian people.

Elective surgery, SMPM score, and the extent of the colorectal neoplasia were related to the long-term survival in the present series. However, only TNM stage was determined to be an independent prognostic factor for survival. The tumor stage being the most determining factor in long-term survival, in line with other studies [12], demonstrates that this population will behave oncologically in a similar way to younger groups of patients with CRC.

In order to identify other factors that delimited the type of elderly patient who would most benefit from surgery in colorectal cancer, the patients were also separated into two groups according to the surgical procedure performed: curative and elective surgery vs palliative and/or emergency surgery. However, neither biodemographic factors nor any preoperative clinical variable could help demonstrate possible differences between these two groups, probably because of the limited number of patients.

The limitations of the study include its retrospective nature, the relatively small size of the series, the unknown proportion of patients with colorectal cancer who were not operated on and were referred for palliative care, and the lack of postoperative quality of life assessment. On the other hand, preoperative geriatric assessment has become a powerful tool that could help to understand what type of patients would benefit the most from the surgical treatment. Due to the retrospective nature of the study design, these data were not evaluated. The strengths of this study include the availability of detailed perioperative information on the oldest population treated in our department, the long follow-up of this cohort, and the hopeful long-term outcomes, which can be extrapolated to the general population of nonagenarians.

## Conclusion

We support that advanced age, per se, is not a disease and should never be a contraindication for a surgical procedure in patients with CRC. Moreover, we consider that chronological age serves as a poor substitute for biological age, which in itself is difficult to define or determine. We conclude that colorectal cancer surgery is relatively safe in selected nonagenarian patients and may achieve a long-term survival.

## Data Availability

Not applicable
